# The value of pre-hospital trauma life support courses for medical personnel—a questionnaire study

**DOI:** 10.3389/fmed.2024.1345310

**Published:** 2024-04-05

**Authors:** Michel Paul Johan Teuben, Nikolaus Löhr, Alba Shehu, Till Berk, Kai Oliver Jensen, Ester Mikova, Martin Brüesch, Stephan Müller, Roman Pfeifer, Ladislav Mica, Hans Christoph Pape, Kai Sprengel

**Affiliations:** ^1^Department of Trauma, University Hospital Zurich, University of Zurich, Zürich, Switzerland; ^2^Institute of Diagnostic and Interventional Radiology, University Hospital Zurich, University of Zurich, Zürich, Switzerland; ^3^University of Bristol Medical School, University of Bristol, Bristol, United Kingdom; ^4^Institute of Anaesthesiology, University Hospital Zurich, University of Zurich, Zürich, Switzerland; ^5^Schutz und Rettung Zürich, Zürich, Switzerland

**Keywords:** pre-hospital trauma life support, training, self-confidence, communication, quality of care, pre-hospital care

## Abstract

**Background:**

The aim of the study was to determine the impact that PHTLS^®^ course participation had on self-confidence of emergency personnel, regarding the pre-hospital treatment of patients who had suffered severe trauma. Furthermore, the goal was to determine the impact of specific medical profession, work experience and prior course participation had on the benefits of PHTLS^®^ training.

**Methods:**

A structured questionnaire study was performed. Healthcare providers from local emergency services involved in pre-hospital care in the metropolitan area of Zurich (Switzerland, Europe) who completed a PHTLS^®^ course were included. Altered self-confidence, communication, and routines in the treatment of severe trauma patients were examined. The impact of prior course participation, work experience and profession on course benefits were evaluated.

**Results:**

The response rate was 76%. A total of 6 transport paramedics (TPs), 66 emergency paramedics (EPs) and 15 emergency doctors (EDs) were included. Emergency paramedics had significantly more work experience compared with EDs (respectively 7.1 ± 5.7 yrs. vs. 4.5 ± 2.1 yrs., *p* = 0.004). 86% of the participants reported increased self-confidence in the pre-hospital management of severe trauma upon PHTLS^®^ training completion. Moreover, according to 84% of respondents, extramural treatment of trauma changed upon course completion. PHTLS^®^ course participants had improved communication in 93% of cases. This was significantly more frequent in EPs than TPs (*p* = 0.03). Multivariable analysis revealed emergency paramedics benefit the most from PHTLS^®^ course participation.

**Conclusion:**

The current study shows that PHTLS^®^ training is associated with improved self-confidence and enhanced communication, with regards to treatment of severe trauma patients in a pre-hospital setting, among medical emergency personnel. Additionally, emergency paramedics who took the PHTLS^®^ course improved in overall self-confidence. These findings imply that all medical personal involved in the pre-hospital care of trauma patients, in a metropolitan area in Europe, do benefit from PHTLS® training. This was independent of the profession, previous working experience or prior alternative course participation.

## Background

The Advanced Trauma Life Support Course (ATLS®) was created in order to educate emergency physicians and improve trauma care within a secondary care setting. Following its success, the need for an equivalent course focusing on the pre-hospital phase of trauma care was identified and the Pre-Hospital Trauma Life Support (PHTLS®) course was proposed (1–3). The PHTLS® course aims to improve the quality of care that (poly)trauma patients receive in the earlier phases of the chain of trauma (2, 4) and consists of a two-day interactive program accompanied by a structured course manual (5). In less developed medical systems, PHTLS® courses have been demonstrated to have a beneficial effect both in terms of participant skills and patient outcome (6–8). However, the benefit of PHTLS® courses in developed high-level medical systems remains relatively unexplored. It has been assumed that even within this context there is a significant improvement in patient outcomes following PHTLS® training.

Demographic studies suggest that accident-related trauma cases are becoming rarer in Western-Europe which decreases the exposure and experience of qualified medical personnel to such cases (9). Structured training concepts such as PHTLS®, which entails theoretical background and intensive team scenario trainings, may compensate for this issue (10). In line with other European countries, various healthcare professionals in Switzerland collaborate to facilitate pre-hospital trauma care (e.g., paramedics, fire fighters and medical doctors) (11–13). The PHTLS® course is open to all first responders as it accommodates for various professions and skillsets, nevertheless it is unclear if a specific group of healthcare providers receives a greater benefit than others. The values gained by well-trained personnel from the training program is still a topic of debate. The aim of the current study was to determine the impact of PHTLS(r) course participation among a heterogeneous group of healthcare professionals on self-confidence, communication, and methodology during the treatment of severe trauma. Additionally, the role of medical profession, work experience, and prior course participation on reported outcome was analyzed.

## Methods

### Ethical approval

This study was reviewed and approved by the local medical ethics committee (protocol no.: KEK-ZH: 2011–0493). This cross-sectional study is in agreement with the recommendations from the Consensus-Based Checklist for Reporting of Survey Studies (CROSS) (14). The CROSS criteria for the current manuscript are provided in Supplementary material 1.

### Participants

Healthcare providers from the local emergency transport service [Schutz & Rettung Zurich (SRZ)] and the University Hospital Zurich (USZ) involved in the pre-hospital care in the metropolitan area of Zurich (Switzerland) were contacted. All participants had successfully completed a PHTLS® course. The SRZ is the largest civil rescue organization in Switzerland with a total of 160 employees who are involved in 24/7 coverage of pre-hospital health care in the area of Zurich.

### PHTLS^®^-course program

The PHTLS® course is a comprehensive program that covers basic theory and skills required to treat trauma victims in a pre-hospital setting. The two-day program addresses key topics and aims to prepare participants to deal accordingly with various trauma scenarios. Simulation and group training exercises are an important component of the program. During the course a systematic approach for trauma care is introduced, and this includes in-field assessment of vitals and the stability of the patient as well as basics of airway management, hemorrhage control and the use of devices and tools to stabilize the patient during the pre-hospital phase and transport. A detailed description of the course program is provided elsewhere (2–4).

### Data collection

A two-page structured questionnaire was sent to all potential participants. The questionnaire included questions aimed at determining the level of experience and previous course participation. Additionally, course participants were asked to determine whether or not improvements in self-confidence and communication were observed after PHTLS® course completion and whether they believed out-of-hospital trauma care had improved as a result. Both open and closed questions were included. The questionnaire was tailor-made and it was decided to keep the questionnaire as compact as possible, in the hope to increase motivation for a participant to complete the whole process. The original version has been provided in Supplementary material 2. Participants were contacted by email twice. No additional in- or exclusion criteria were defined as the questionnaires have been send to the target group only. All fully filled out questionnaires were used for the final analysis. Data was extracted from all fully filled out and returned questionnaires and data has been stored anonymous. No data-imputation techniques have been applied.

### Study groups

For the purpose of the study the following study groups were defined:

Group TP: transport paramedics (incl. firefighters)

Group EP: emergency paramedics/nurses

Group EMD: emergency medical doctors

Please see Supplementary material 3 for a brief overview of the regular training program of all previously mentioned positions.

### Outcome parameters

#### Primary outcome

Difference between groups in reported altered self-confidence in the pre-hospital treatment of severe trauma patients.

#### Secondary outcomes

1. Differences between groups in reported improved communication in the pre- and in-hospital setting
2. Subjective impact of PHTLS® course participation on execution of pre-hospital trauma care
3. Identification of predictive factors for improved self-confidence in the pre-hospital treatment of severe trauma patients.

### Statistical analysis

Completed questionnaires were analyzed and statistical interpretation was performed using SPSS (Version 22.0, IBM Inc., Armonk, USA). Percentages were calculated in relation to the response to each question where applicable. Parameters between groups were compared with *T*-tests or Mann–Whitney’s *U*-test for continuous variables and Chi-square or Fisher’s Exact Test for categorical variables. *p*-values <0.05 were considered statistically significant.

Furthermore, in order to determine which factors predicted improved self-confidence in the pre-hospital treatment of severe trauma patients, a backward stepwise logistic regression analysis was performed. Prior to executing the multivariable analysis, all factors with a positive *p*-value of less than 0.3 were selected from a univariable comparison between two groups; participants with reported improvement of self-confidence in the pre-hospital treatment of trauma cause vs. those participants without reported improvement. To do so, a backward stepwise logit regression analysis was performed and the model was validated by a forward regression analysis.

## Results

A total of 115 potential participants were contacted, of whom 8 qualified as transport paramedics (TP), 82 were emergency paramedics (EP) and 25 worked as emergency medical doctors (EMD). A total of 87 healthcare professionals who completed interviews were eligible for analysis leading to an overall response rate off 76%. Further breakdown shows 66/82 emergency paramedics, 15/25 emergency medical doctors, 6/8 transport paramedics participated.

31 participants were involved in the PHTLS® course prior to 2008, whereas 47 individuals completed the course between 2008 and 2015. Nine of the participants did not declare when they participated in the course. Overall years of experience in the field of pre-hospital emergency care prior to course participation was 6.7 ± 5.2 years. The TP-group had an average of 5.8 ± 1.7 years of experience, whereas the EP-group had 7.1 ± 5.7 years, and the EMD-group had 4.5 ± 2.0 years. Emergency paramedics have significantly more working experience than emergency physicians (*p* = 0.004). The following additional courses have been completed by the participants prior to PHTLS® training: Advance Medical Life Support (AMLS®) (*n* = 48), Megacode (*n* = 20), Advanced Trauma Life Support (ATLS®) (*n* = 17) and Advanced Cardiac Life Support (ACLS®) (*n* = 5). Other courses completed included: European Pediatric Advanced Life Support (EPALS) and the Pediatric Advanced Life Support (PALS). Characteristics of participants of different study groups are shown in Table 1, no comparison of specific course participation between groups was performed.

**Table 1:** Overview of prior working/training experience of participants.

	Transport paramedic *N = 6*	Emergency paramedic *N = 66*	Emergency physician *N = 15*
Prior working experience (yrs)	5.8 ± 1.7	7.1 ± 5.7*	4.5 ± 2.0*
No prior course participation (%)	50%	3%	0%
Prior course participation			
AMLS®	0	48	**0**
Megacode	3	13	4
ATLS®	0	6	11
ACLS®	0	5	0
Other	0	11	2

All data in mean (std). Statistically significant outcome: *p = 0.004 (Gr. Emergency paramedic vs. emergency physician for parameter prior working experience in years. Other comparisons did not show any statistically significance). No comparison of specific course participation was made between groups. AMLS®, Advanced medical life support; ATLS®, Advanced trauma life support; ACLS®, Advanced cardiac life support.

### Impact of PHTLS^®^-training on treatment of severe trauma patients among health care professionals

86% of the participants experienced an increase in self-confidence in treating severe trauma patients in the pre-hospital setting after PHTLS® course completion. No differences in the degree of the subjective improvement in confidence was found between groups.

In addition to this, 84% of the participants stated the PHTLS® training resulted in altered methodology of pre-hospital care of polytraumatized patients. Although, no differences were seen between groups.

### Increased quality of communication after PHTLS^®^-course participation

Following the completion of the course, a total of 81 out of 87 participants (93%) reported an improvement in inter-professional communication skills utilized during the pre-hospital treatment of polytrauma patients. Emergency paramedics reported significantly more improved pre-hospital communication in comparison to participants from the transport personnel-group (respectively 97% vs. 67%, *p* = 0.03).

Out of all respondents, 77% believed that the communication between the pre-hospital team and the trauma bay team improved after PHTLS® completion. No statistically significant differences between groups were seen. Personnel-specific responses and outcome are summarized and compared in Table 2.

**Table 2:** Reported impact of PHTLS^®^ course participation among groups.

Post-PHTLS^®^	Transport paramedic *N = 6*	Emergency paramedic *N = 66*	Emergency physician *N = 15*
Enhanced self-confidence	67%	89%	73%
Altered pre-hospital treatment of polytrauma	67%	86%	73%
Improved pre-hospital communication	67%*	97%*	87%
Improved in-hospital communication	83%	76%	73%

Pre-hospital communication: internal communication of the pre-hospital team. In-hospital communication: communication between the pre-hospital team and the trauma bay team. Statistically significant outcome (transport paramedic vs. emergency paramedic for the parameter improved pre-hospital communication): *p = 0.03.

### Independent predictors for unaltered self-confidence in treating polytrauma cases after course completion

After univariable analysis of potential predictors for improved self-confidence among course participants, the following factors were selected for multivariable regression analysis: all specific profession types and prior ATLS®-course participation. The regression analysis identified the profession of emergency paramedic as the single independent predictor for improved self-confidence after PHTLS® course participation. Previous working experience or alternative course participation was not associated with improved self-confidence. Results of univariable analysis as well as the regression analysis are displayed in Tables 3, 4.

**Table 3:** Univariable analysis of participants characteristics and improved self-confidence upon PHTLS^®^ course participation.

	Improvement post-PHTLS^®^	Unaltered post-PHTLS^®^	*p*-value
Working experience (yrs)	6.5 (5.4)	7.1 (4.1)	0.71
Profession			
Transport paramedic	67%	33%	**0.22**
Emergency paramedic	89%	11%	**0.07**
Emergency physician	73%	27%	**0.23**
Prior courses			
Megacode	80%	20%	0.46
AMLS®	88%	12%	0.76
ATLS®	77%	23%	**0.24**
ACLS®	80%	20%	0.54
Other	92%	8%	0.68

Data in mean (std). All bold factors have been selected for multivariable analysis. AMLS®, Advanced medical life support; ATLS®, Advanced trauma life support; ACLS®, Advanced cardiac life support. The parameters extracted from the univariable analysis (table 3) and utilized for multivariable analysis (table 4) are being marked by a bold p-value.

**Table 4:** Results of stepwise logit regression analysis for improved self-confidence upon PHTLS^®^ course participation.

	Odds ratio	Lower 95% C.I.	Upper 95% C.I.	*p*-value
Emergency paramedic	3.93	1.109	13.944	0.034
Chi^2^ (df = 1)	4,361			

## Discussion

This study revealed that successful PHTLS® course completion is associated with improved self-confidence when treating severe trauma patients in the pre-hospital setting among medical emergency personnel in a European metropolitan area. The profession of emergency paramedic, rather than working experience or alternative course participation, was predictive for increased self-confidence. In addition, PHTLS® training resulted in improved communication among healthcare personnel as well as altered pre-hospital care pathways.

The results imply that healthcare professionals in well-developed regions, regardless of background, may benefit from PHTLS® training as both self-confidence and quality of communication noticeably improved after course participation. This is independent of extensive working experience and prior completion of other trauma courses. Based on the multivariable analysis performed the profession that benefits most from the completion of the course is emergency paramedics.

The findings of the current study show that PHTLS® course completion can be linked to the improvement of self-confidence when managing pre-hospital trauma patients and the overall quality of communication. Studies have shown PHTLS® courses provide various additional benefits. Johansson et al. demonstrated that PHTLS® training of Swedish ambulance personnel is associated with reduced mortality (11). Similar improvements in mortality have been described in less developed countries (6–8). A study from Haeske et al., reported quality of documentation was improved upon PHTLS® course participation (5). The current study along with the information reported by Haeske et al. both demonstrate a real impact of the PHTLS course leading to healthcare professionals altering real patient care upon course completion (5). This is further supported by a study from Ali et al. who reported that pre-hospital trauma care was altered, specifically by more frequent airway control, oxygen suppletion, C-spine control, hemorrhage management and fracture splinting (15).

Based on a previous studies, it is tempting to hypothesize that there are superior outcomes relating to improved efficacy of care in trauma pre-hospital care upon the completion of PHTLS® training (5). Increased efficiency due to PHTLS® training may partly be explained by increased clinical and management skills (Ali et al.) (16). Increased self-confidence and better communication, as found in the current study, most likely also contribute to the overall improvement in pre-hospital care.

It has been previously demonstrated, that in addition to real-life experience, training programs/courses have the potential to boost self-confidence in performing life-saving interventions in trauma (17). Although, in line with Haeske et al., it was found that the quantitative amount of real-life working experience did not affect the impact course participation had on altered self-confidence in pre-hospital trauma care (18). The findings of the current study further suggest that prior participation in other official (trauma) courses does not influence reported changes in subjective improvement upon PHTLS® course completion. Even though, certain factors are not influenced by course participation, this investigation implies that the PHTLS® course is advantageous for all health care professionals. Hence this training should be considered as complementary to alternative (trauma) courses (19, 20). Of note, as there is overlap between the PHTLS®-training and other courses, the specific impact of PHTLS® itself cannot be extracted by the current study as no other intervention group has been included in this study (21). Theoretically, post-course improvement can be expected with other courses than PHTLS® as well.

The current study has several limitations. Firstly, it is a subjective self-reported evaluation of the impact of PHTLS® course participation, rather than a test of pre-hospital care competence. Nevertheless, self-assessments are an adequate tool to determine alterations in quality of care (22). Secondly, the benefit of course participation in (para)medical students was not studied, as only medical professionals with relevant working experience have been contacted. Future studies should focus on the potential role of the PHTLS® course in the education/curriculum of (para)medical students. Besides, no detailed information on timing and exact content of prior course participation was collected. Furthermore, the time interval between course participation and survey participation differs between participants. Future follow-up studies are indicated to analyze the development of self-confidence and communication skills among medical emergency personnel over time, as well as the need for PHTLS®-refresher courses.

## Conclusion

The current study reveals that PHTLS® training is associated with improved self-confidence and enhanced communication among medical emergency personnel in a European metropolitan area when treating severe trauma patients in the pre-hospital setting. The profession of medical paramedic, rather than working experience or alternative course participation, was predictive for increased self-confidence after PHTLS® course participation. In conclusion, it can be stated that medical personal involved in the pre-hospital care of trauma patients, in a metropolitan area in Europe, can benefit from PHTLS® training. This is independent of the persons function (transport/emergency paramedic or medical physician), previous working experience or prior alternative course participations.

## Data availability statement

The raw data supporting the conclusions of this article will be made available by the authors, without undue reservation.

## Ethics statement

The studies involving humans were approved by Zurich University Hospital medical ethics committee (protocol no.: KEK-ZH: 2011-0493). The studies were conducted in accordance with the local legislation and institutional requirements. Written informed consent for participation was not required from the participants or the participants’ legal guardians/next of kin in accordance with the national legislation and institutional requirements.

## Author contributions

MT: Writing – original draft, Writing – review & editing. NL: Writing – original draft, Writing – review & editing. AS: Writing – original draft, Writing – review & editing. TB: Writing – review & editing. KJ: Writing – original draft, Writing – review & editing. EM: Writing – review & editing. MB: Writing – original draft, Writing – review & editing. SM: Writing – original draft, Writing – review & editing. RP: Writing – original draft, Writing – review & editing. LM: Writing – review & editing. HP: Writing – review & editing. KS: Writing – review & editing.
